# Nano-socketed nickel particles with enhanced coking resistance grown *in situ* by redox exsolution

**DOI:** 10.1038/ncomms9120

**Published:** 2015-09-11

**Authors:** Dragos Neagu, Tae-Sik Oh, David N. Miller, Hervé Ménard, Syed M. Bukhari, Stephen R. Gamble, Raymond J. Gorte, John M. Vohs, John T.S. Irvine

**Affiliations:** 1 School of Chemistry, University of St Andrews, St Andrews, KY16 9ST Scotland, UK; 2 Department of Chemical and Biomolecular Engineering, University of Pennsylvania, Philadelphia, Pennsylvania 19104, USA; 3 Sasol Technology (UK) Ltd., St Andrews, KY16 9ST Scotland, UK

## Abstract

Metal particles supported on oxide surfaces are used as catalysts for a wide variety of processes in the chemical and energy conversion industries. For catalytic applications, metal particles are generally formed on an oxide support by physical or chemical deposition, or less commonly by exsolution from it. Although fundamentally different, both methods might be assumed to produce morphologically and functionally similar particles. Here we show that unlike nickel particles deposited on perovskite oxides, exsolved analogues are socketed into the parent perovskite, leading to enhanced stability and a significant decrease in the propensity for hydrocarbon coking, indicative of a stronger metal–oxide interface. In addition, we reveal key surface effects and defect interactions critical for future design of exsolution-based perovskite materials for catalytic and other functionalities. This study provides a new dimension for tailoring particle–substrate interactions in the context of increasing interest for emergent interfacial phenomena.

## Abstract (web summary)

Metal particles supported on oxide surfaces are widely used catalysts, and the composites are generally formed by deposition or exsolution methods. Here, the authors show that nickel particles exsolved from the parent perovskite exhibit enhanced stability due to a stronger metal–oxide interface.

## Graphical abstract

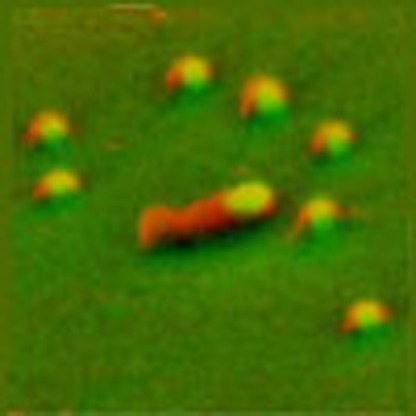

Several particle characteristics, including size and morphology, but most importantly interaction with the oxide support determine the activity, selectivity and stability of supported metal catalysts; thus, controlling these aspects is essential for both fundamental and applicative reasons^1^,^2^,^3^. The vast majority of supported particles are prepared by deposition methods (for example, infiltration, Supplementary Fig. 1a), which although widely applicable, provide limited control over particle interaction with the support, during deposition and over time^4^,^5^. This leads to deactivation by agglomeration^5^ or by coking (carbon accumulation on the metal in hydrocarbon environment) in industrially critical processes such as syngas production by methane steam reforming^6^,^7^. Several post-particle growth procedures have been developed to delay agglomeration, by partly embedding or fully encapsulating the particles in thin oxide layers^8^,^9^, while coking may be diminished by mild conditioning or alloying^10^, although these intricate solutions may be temporary or compromise activity.

Previous studies demonstrated that catalytically active transition metals can be substituted on the B-site of perovskite oxides (ABO_3_), in oxidizing conditions, and released (exsolved) on the surface as metal particles following reduction (Supplementary Fig. 1b), with applications in catalysis ranging from automotive emission control to solid oxide fuel/electrolysis cells^11^,^12^,^13^,^14^,^15^,^16^,^17^. Interestingly, several reports find exsolved particles to be more resilient to agglomeration and coking as compared to deposited analogues, although the origin of this stability is unclear^11^,^18^.

Here we reveal that this stability is due to exsolved particles being partially embedded in the surface of a parent perovskite and thus exsolution may be regarded as an elegant one-step environmentally friendly method to grow pinned, coking-resistant, socketed particles. We also provide critical insights into surface effects and defect interactions relevant for the future development of exsolution process but also for perovskite bulk or surface related applications.

## Results

### Surface effects controlling exsolution

In this work, we employ compositions derived from SrTiO_3_, an archetype oxide of considerable interest for applications ranging from solid oxide fuel cells to complex oxide electronics^19^,^20^,^21^,^22^. We introduce A-site deficiency, La_*x*_Sr_1−3*x*/2_TiO_3_ (*x*=0.4), which we have shown to promote B-site redox exsolution^14^,^17^. We use modest Ni^2+^ doping levels, La_0.4_Sr_0.4_Ni_*y*_Ti_1−*y*_O_3−*y*_ (*y*=0.03) or La_0.4+2*x*_Sr_0.4-2*x*_Ni_*x*_Ti_1−*x*_O_3_ (*x*=0.06), to improve relevance to other systems where low loadings are desirable due to cost and/or low solubility in the host lattice (for example, noble metals).

**Figure 1:**
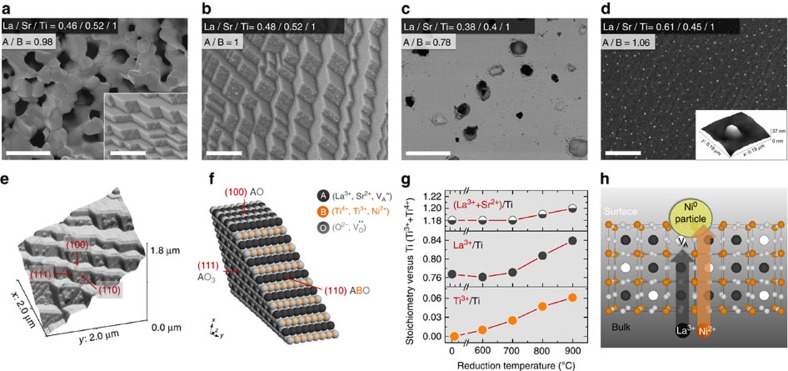
Surface effects controlling exsolution. SEM micrographs of the native surface of a porous La_0.4_Sr_0.4_Ni_0.03_Ti_0.97_O_3−*γ*_ sample (**a**) before (scale bars, 50 μm (overview); 1 μm (detail)) and (**b**) after reduction (5% H_2_/Ar, 930 °C, 20 h); scale bar, 1 μm. SEM micrographs of the polished surface of a 94% dense La_0.4_Sr_0.4_Ni_0.03_Ti_0.97_O_3−*γ*_ pellet (**c**) before (scale bar, 50 μm) and (**d**) after reduction (5% H_2_/Ar, 900 °C, 20 h); the inset depicts a three-dimensional (3D) AFM image of a particle; scale bar, 1 μm. (**e**) 3D AFM reconstruction of a native surface similar to **a** and **b** highlighting the calculated orientations of the facets (see Supplementary Fig. 6 for details); (**f**) atomic scale model highlighting the orientation and probable termination layers of the terraces found in samples **a**, **b** and **e**. (**g**) Surface composition versus reduction temperature by *in situ* XPS, carried out on a sample with nominal composition La_0.52_Sr_0.28_Ni_0.06_Ti_0.94_O_3_, in 5% H_2_/Ar, using 0.5 h isotherms (see Supplementary Fig. 8 and Supplementary Table 1 for the corresponding XPS spectra and analysis, respectively). (**h**) Schematic of the key processes occurring during the reduction of an A-site-deficient surface such as **c**, highlighting that Ni^2+^ and La^3+^ diffuse in parallel from the bulk to the surface, forming Ni particles, and filling available A-site vacancies, respectively. The ratios in (**a**–**d**) indicate surface (2–10 nm) stoichiometry from XPS (error±0.01 versus Ti; the corresponding spectra is given in Supplementary Fig. 3).

To form the oxide supports with given stoichiometry into microstructures relevant for applications (usually in flat dense, porous or powder form), the oxides have to be exposed to synthetic- or processing-specific conditions that may cause the surface stoichiometry to deviate as compared with the nominal bulk (that is, ‘native surface'). For example, for porous La_0.4_Sr_0.4_Ni_0.03_Ti_0.97_O_3−*γ*_, while the bulk displayed a quasi-cubic perovskite structure (Supplementary Fig. 2) with an A/B ratio close to the nominal value of 0.8 (La_0.4_Sr_0.4_Ni_0.03_Ti_0.97_O_3−*γ*_≡‘A_0.8_BO_3−*γ*_', see Fig. 1c), the native surface showed a high A/B value of ∼0.98 (Fig. 1a). Perovskite oxides are known to develop A-site overstoichiometric surfaces (‘A_1+*α*_BO_3+*γ*_'), probably as a result of cation size mismatch and charge compensation^23^, with detrimental effects on certain catalytic processes, such as the oxygen reduction reaction^24^. Surface A-cation enrichment is also expected to hinder or supress exsolution since we have found previously that A-site stoichiometric formulations are less prone to exsolve from the B-site as compared with A-site-deficient ones^14^. The surface excess is thought to be accommodated as perovskite-type Ruddlesden-Popper structures or AO islands^25^,^26^. Here the A/B ratio is just below unity, suggesting the bulk perovskite structure persists up to the surface, consistent with transmission electron microscopy (TEM) observations, but the surface itself has higher A-site occupancy as compared with the bulk (Supplementary Fig. 4). This implies that surface A-site vacancies are unfavourable and will be naturally filled given sufficient temperature, but also that A-site-deficient formulations may be used to minimize surface A-site cation segregation where this is regarded to be detrimental.

An important feature is that several perovskites, and possibly other oxides used as supports, may develop faceted surfaces, as exemplified in Fig. 1a, inset, suggesting that surfaces can be spatially inhomogeneous as well. This is revealed unquestionably through reduction, which triggers particle growth preferentially on certain facets (Fig. 1b). By contrast, a surface of nominal stoichiometry, which can be exposed by polishing away the native surface or by cleaving through the bulk, grows particles much more uniformly (Fig. 1d; Supplementary Fig. 5). Atomic force microscopy (AFM) surface reconstruction carried out on a representative faceted surface with exsolved particles, allowed us to measure the angles between different facets (Supplementary Fig. 6), and show that they correspond to different perovskite orientations, as highlighted in Fig. 1e. On the basis of this, TEM of near surface region (Supplementary Fig. 4), and the surface composition derived from X-ray photoelectron spectroscopy (XPS), the facets with smooth appearance in Fig. 1a,b, corresponds to A-site terminated (100) and (111) orientations, while the ones with rough morphology correspond to ABO^4+^/O_2_^4−^ terminated (110) orientations, as illustrated in Fig. 1f. On the basis of our previous observations, exsolution occurs when the oxygen vacancy concentration induced through reduction reaches a sufficiently high concentration (*δ*_lim_) in the presence of A-site vacancies (*α*) such that the perovskite lattice becomes destabilized by high deficiency on two of its three primitive sites and will spontaneously exsolve from the B-site to re-establish stoichiometry^14^:


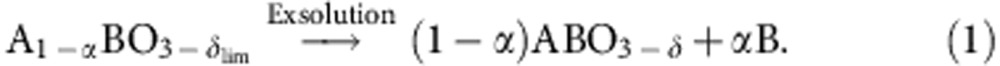


**Figure 2:**
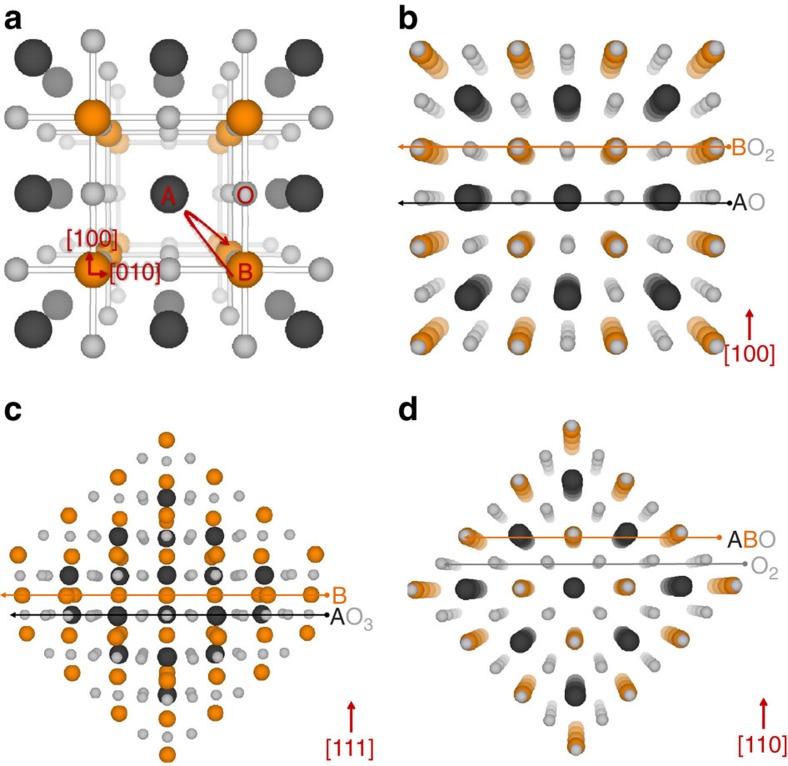
Possible orientations and termination layers for a cubic perovskite crystal ABO_3_. Depending on the direction from which it is viewed, the perovskite crystal structure (**a**) may be described as alternating planes of (**b**) AO and BO_2_ in the [100] direction; (**c**) B and AO_3_ planes in the [111] direction; (**d**) ABO and O_2_ planes in the [110] direction. The curved arrow in **a** illustrates the diffusion trajectory of B-site cations down the [001] direction, according to De Souza *et al*^27^.

Therefore, the systematic presence of particles on (110) terminations is unlikely to be incidental, and may be related to the fact that this is the only orientation in this quasi-cubic crystal in which all primitive perovskite sites holding the key defects required for exsolution are coplanar (Figs 1f, 2) and thus potentially in favourable proximity to nucleate particles, on a surface that is overall quasi-stoichiometric and thus not prone to exsolve (Fig. 1b). Nucleation on (110) surfaces may also be facilitated by their rough morphology as compared with (100) and (111) (see Fig. 1a,b), since the nucleation barrier is generally lowered by the presence of crystal defects.

### Insights into bulk processes from surface effects

It should also be noted that the preferential exsolution of particles on (110) terminations could also represent a reflection of bulk processes. According to De Souza *et al*.^27^, B-cation migration in perovskites is likely to occur between adjacent B-sites along a curved trajectory in the (110) planes, and is substantially facilitated by the presence of A-site vacancies due to lowering of migration repulsions. Figure 2a illustrates a view of the perovskite lattice in the direction of migration, down the cubic [001] direction (additional projections are given in Supplementary Fig. 7). Thus, it seems that (110) planes are particularly suitable for B-site cation diffusion and the abundance of A-site vacancies in our systems may well facilitate the process, supplying exsolvable species to the surface and providing additional reasons as to why A-site-deficient perovskites were found to be much more effective towards exsolution as compared with their stoichiometric analogues^14^.

Further insight into the diffusion processes occurring during exsolution may be extracted from the XPS data in Fig. 1. While native surfaces are largely unchanged following exsolution reflecting their stable, yet rigid nature (Fig. 1a versus b), surfaces of nominal (A-site deficient) stoichiometry show an enrichment of A-site cations, in particular La^3+^ (Fig. 1c versus d), perhaps shedding light on the transport processes occurring inside the bulk. Similar cation transport to the surface was observed by *in situ* XPS (Fig. 1g; Supplementary Fig. 8; Supplementary Table 1), which shows that as the reduction temperature increased, the perovskite was reduced to higher extents (indicated by an increase in Ti^3+^), and in parallel the A/B and La/Ti ratios also increased. The exsolution of Ni particles was observed by XPS and scanning electron microscope (SEM), but quantification was not included in Fig. 1g, due to significant errors associated with low Ni doping level and overlap with the La peak. Thus, during exsolution on bulk-like surfaces nickel diffuses from the bulk to the surface to form particles, while in parallel La^3+^ (and Sr^2+^ to less extent) gradually fills surface A-site vacancies (Fig. 1h). The filling of surface vacancies, eventually leading to a newly formed ‘rigid' ‘native surface', is expected to gradually limit lanthanum and nickel diffusion from the bulk to the surface, and thus exsolution, and may possibly contribute to locking the particles into place.

By mapping Ni particles in Fig. 1d to estimate the total amount exsolved, it becomes apparent that the Ni must have diffused from ∼100nm deep in the bulk, confirming that B-site transport to the surface is critical for exsolution. Moreover, this Ni amount is in similar quantity to the La^3+^ that enriched the surface during reduction, as determined by XPS (see Supplementary Fig. 9 and Supplementary Note 1 for particle analysis and quantification, respectively), suggesting that B-site and A-site diffusion are probably correlated, possibly as tungsten bronze ephemeral units (Supplementary Fig. 10). Notably, the different mobility of A-site cations during reduction shows that their nature may directly influence B-site diffusion and hence exsolution, providing another dimension to tailor it. Indeed, La facilitating exsolution is consistent with our previous findings by which compositions with high La/Sr ratios produced more numerous and better distributed particles^14^, which may provide a conceptual approach for overcoming the rigidity of the native surface in exsolution, as exemplified in Supplementary Fig. 11 for La_0.46_Sr_0.34_Ni_0.03_Ti_0.97_O_3_, which displayed improved native surface particle coverage compared with La_0.4_Sr_0.4_Ni_0.03_Ti_0.97_O_3−*γ*_ (Fig. 1b).

### Particle–substrate interface

**Figure 3:**
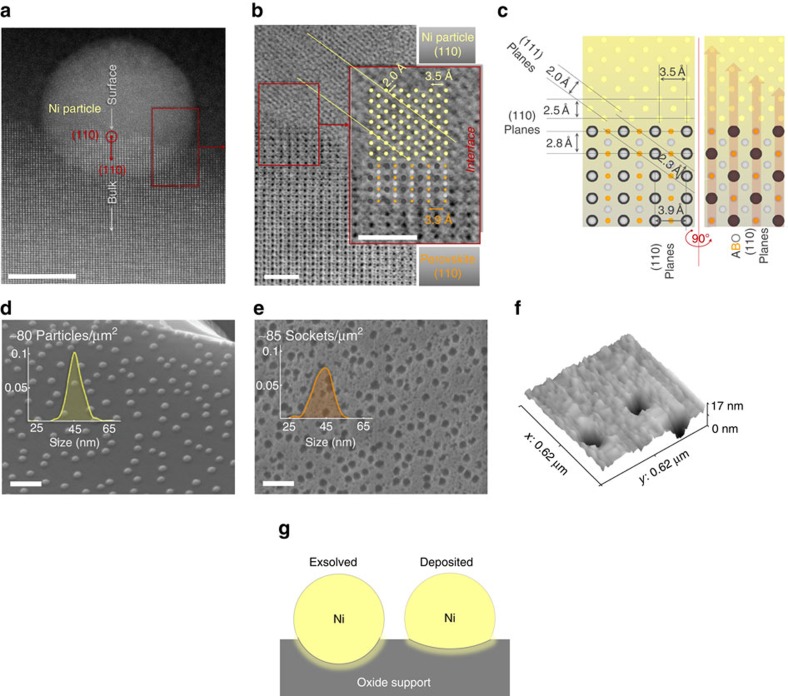
Exsolved particle–substrate interface. (**a**) TEM micrograph (dark field) of a Ni particle exsolved on (110) native surface facet (see Fig. 1b) after ageing (∼3% H_2_O/5% H_2_/Ar, 930 °C, 60 h); scale bar, 10 nm. (**b**) TEM micrograph detail (bright field) of the metal–perovskite interface highlighting the corresponding atomic planes and orientations; scale bars, 1 nm. (**c**) Schematic atomic model of the metal–perovskite orientation relationship based on **b**. SEM micrographs of exsolved Ni particles (La_0.52_Sr_0.28_Ni_0.06_Ti_0.94_O_3_, 5% H_2_/Ar, 920 °C, 12 h) (**d**) before and (**e**) after etching particles in HNO_3_; the insets show the size histogram of the particles and sockets, respectively, as determined through image analysis; scale bars, 200 nm. (**f**) Three-dimensional AFM of sockets similar to those in **e**. (**g**) Schematic illustration of the particle–substrate interface for deposited and exsolved nickel particles.

TEM imaging of the particles exsolved on (110) terminations revealed that when the bulk is also aligned to [110], this corresponds to a profile view of the interface, the particle consists of a slightly oblate spheroid, ∼30% submerged into the parent oxide surface (Fig. 3a; Supplementary Fig. 12). The fact that particles are indeed universally socketed in the surface was confirmed by etching them in concentrated HNO_3_, which left behind pits with similar number density and size distribution as the particles themselves (Fig. 3d,e). An AFM detail of an etched surface is shown in Fig. 3f demonstrating they are indeed embedded to a considerable depth in the parent oxide support. Figure 3a,b shows that the bulk ‘cubic' perovskite structure appears to be retained all the way to the surface and to the metal–oxide interface, which is smooth and continuous. Particles are metallic in nature and epitaxial with respect to the parent oxide as determined by electron diffraction (Supplementary Fig. 13). The epitaxic relationship is highlighted in Fig. 3b which depicts the perovskite (111) planes aligned to the (111) planes of the Ni lattice. A more detailed analysis of the proposed (ideal) orientation relationship is given in Fig. 3c, although it should be noted that dislocations are likely to occur in the metal lattice (possibly visible in Fig. 3b) to accommodate the lattice mismatch (the cell parameters for the perovskite and nickel lattices are ∼3.9 and ∼3.5 Å, respectively). In addition, the fact the metal lattice is growing from the oxide lattice might naturally facilitate interdiffusion between the two, which has been shown to significantly increase adhesion between metal and oxide phases even when occurring over 1–2 unit cell thin interfaces^28^. Overall, these features appear to contribute synergistically towards considerably improving the anchorage of exsolved particles, explaining both our current observations and those described previously^18^. By contrast, little to no particle embedding could be observed by AFM in samples prepared by conventional deposition on similar A-site-deficient perovskites (see Supplementary Fig. 14), indicating a much smaller degree of interaction occurred during growth between the deposited metal and oxide phases.

### Implications of particle–substrate interactions

**Figure 4:**
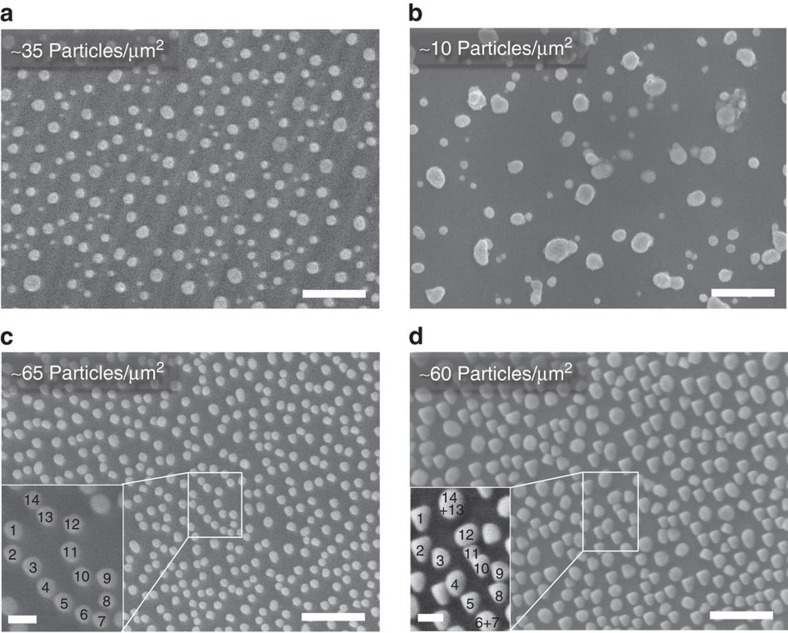
Thermal stability of deposited and exsolved Ni particles. SEM micrographs of vapour-deposited Ni particles on La_0.4_Sr_0.4_TiO_3_ (**a**) before and (**b**) after ageing (H_2_, 650 °C, 24 h and 800 °C, 6 h). SEM micrographs of Ni particles exsolved from cleaved bulk surface of La_0.52_Sr_0.28_Ni_0.06_Ti_0.94_O_3_ (5% H_2_/Ar, 900 °C, 12 h) (**c**) before and (**d**) after ageing (5% H_2_/Ar, 900 °C, 70 h). Scale bars, 500 nm (overview), 100 nm (detail).

The profound morphological differences that distinguish exsolved particles from the deposited analogues, highlighted above and summarized in Fig. 3g, are clearly reflected by their stability and functionality. Exsolved Ni particles display considerably lower tendency to agglomerate and coke as opposed to deposited ones, as illustrated below for various scenarios. Figure 4 compares the thermal stability of deposited and exsolved particles of similar size showing that while the former coalesce rapidly at 800 °C or below (Fig. 4a,b), the latter are reasonably stable over tens of hours at 900 °C, in spite of having almost double initial particle loading (Fig. 4d). Examination of Fig. 4b,d reveals that the size of exsolved particles does increase over time possibly because of additional exsolution from the bulk and to less extent due to coalescence since ∼90% of the particles are preserved throughout the ageing test. Coalescence seems to occur predominantly when particles were initially tangent or in close proximity to each other (see inset of Fig. 4d). Nonetheless, exsolved particles generally behave as if pinned to their original location, showing a level of stability beyond metal–oxide interfaces produced through conventional means on a similar A-site-deficient perovskite.

**Figure 5:**
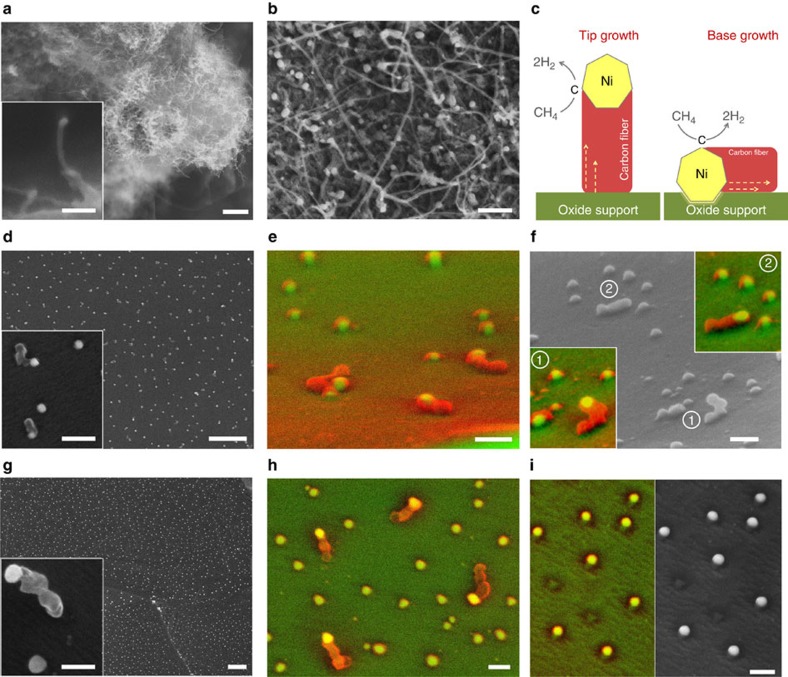
Emergent anti-coking trait of exsolved particles. (**a**) Approximately 20 nm Ni particles formed by infiltration on La_0.4_Sr_0.4_TiO_3_ (see Supplementary Fig. 15a for initial microstructure) after coking test, showing significant carbon fibre growth; scale bars, 1 μm (overview); 100 nm (detail). (**b**) Ni particles (30–100 nm) prepared by vapour deposition on La_0.4_Sr_0.4_TiO_3_ (see Supplementary Fig. 15b for initial microstructure) showing considerable carbon fibre growth; scale bar, 0.5 μm. (**c**) Schematic of possible carbon fibre growth mechanisms based on refs 6, 7, 29. (**d**) Approximately 25 nm Ni particles formed by exsolution from La_0.52_Sr_0.28_Ni_0.06_Ti_0.94_O_3_ (5% H_2_, 880 °C, 6 h), after coking test, showing limited carbon fibre growth; scale bars, 0.5 μm (overview); 100 nm (detail). (**e**) False colour micrograph depicting a side view detail of sample (**d**); scale bar, 100 nm. (**f**) Side view micrograph and false colour micrograph detail insets of different region in sample **d**; scale bar, 100 nm. (**g**) Approximately 60 nm Ni particles formed by exsolution from La_0.52_Sr_0.28_Ni_0.06_Ti_0.94_O_3_ (5% H_2_/Ar, 1,000 °C, 6 h) after coking showing limited coking; scale bars, 1 μm (overview); 100 nm (detail). (**h**) False colour top view micrograph of sample **g**; scale bar, 100 nm. (**i**) False colour and corresponding secondary electron micrographs of a different region in sample **g** showing particles alongside empty sockets; scale bar, 100 nm. In all cases, the coking test was carried out in 20% CH_4_/H_2_, at 800 °C, for 4 h. In the false colour micrographs, green was used for the perovskite, red for carbon and yellow for Ni metal.

The second consequence of particle–substrate interaction studied here is in relation to the tendency of Ni to grow carbon fibres in a hydrocarbon environment, which is detrimental in various applications, as explained before. Ni metal particles are well-known to catalyse carbon fibre formation in a wide range of particle sizes and hydrocarbon-containing environments. To test coking stability, samples were exposed to 20% CH_4_/H_2_, at 800 °C, for 4 h. Consistent with previous reports, relatively small Ni particles (∼20 nm) prepared by infiltration on La_0.4_Sr_0.4_TiO_3_ (Supplementary Fig. 15a) coked severely, showing well developed fibres, as illustrated in Fig. 5a (refs 6, 7). Similarly, Ni particles prepared by vapour deposition on La_0.4_Sr_0.4_TiO_3_ with particle size within 30–100 nm range (see Supplementary Fig. 15b for initial microstructure and size distribution) also produced large amounts of micron-long carbon fibres, as shown in Fig. 5b. Notably, exsolved Ni metal particles of comparable sizes, ∼25, 60 and 80 nm, displayed considerably less carbon fibre growth, as illustrated in Fig. 5d,g and Supplementary Fig. 15d, respectively. Carbon fibre growth on Ni particles has been generally shown to occur in a characteristic manner, by a so-called ‘tip-growth'^7^ mechanism illustrated in Fig. 5c (left). In this mechanism, exhibited by the deposited Ni particles (see Fig. 5a,b), carbon initially dissolves into the Ni lattice, while the fibre grows at the metal particle–oxide support interface, resulting in particle uplifting from its original location. Most likely, the remarkable decrease in the tendency to grow carbon fibres observed in the exsolved Ni systems is due to the strong interaction between the exsolved socketed particle and the parent oxide support which prevents particle uplifting and subsequent fibre growth. Limited carbon fibre formation may also be found occasionally in the exsolved Ni samples (see Fig. 5d–h), although it should be noted that in this case the fibres were considerably shorter than those found in the deposited Ni samples (compare Fig. 5b with Fig. 5g,h). It should also be noted that for the ∼25 nm exsolved Ni particles most of the short carbon fibres that form appear to be laying on the oxide support (see Fig. 5e) rather than standing perpendicular on it as expected for a tip-growth mechanism. This type of carbon fibre growth is reminiscent of a so-called ‘base growth' mechanism where the particle remains attached to the substrate while the fibre grows on top of it (see Fig. 5c, right). Interestingly, base growth is generally thought to occur particularly when particles adhere strongly to the support^29^, which seems to provide additional proof towards the argument that exsolved particles possess superior adhesion to the parent substrate, which in turn prevents particle uplifting. Nonetheless, close examination of this sample reveals that particle uplifting may still occur occasionally (area 1 in Fig. 5f) alongside seemingly base growth (area 2 in Fig. 5f). Similar behaviour was found for the ∼60 nm exsolved Ni particle system, which also displayed remarkably low extent of coking with limited formation of relatively short carbon fibres, as shown in Fig. 5g,h. Interestingly, in this sample, a few areas displaying empty sockets alongside metal particles were also observed (see Fig. 5i), indicating that probably for this particle size range carbon growth by particle uplifting may start to dominate. Carbon growth by particle uplifting seems to occur exclusively for the ∼80nm exsolved particle also (Supplementary Fig. 15d), perhaps indicating that the interaction and adhesion between exsolved and parent oxide diminish with increasing particle size.

**Figure 6:**
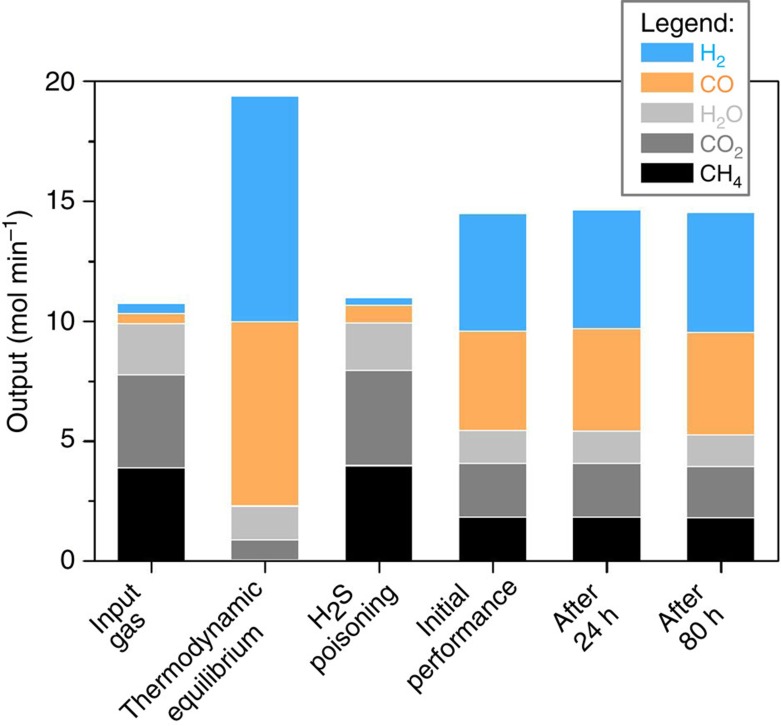
Reforming test on a La_0.52_Ca_0.28_Ni_0.06_Ti_0.94_O_3_ perovskite powder with exsolved Ni particles. The powder (∼1 m^2^ g^−1^ total surface area) was reduced in the testing setup at 900 °C, for 4 h in 5% H_2_/Ar before the reforming test, to exsolve Ni particles on the surface. The reforming test was carried out at 900 °C, and the input gas consisted of: 4% H_2_, 4% CO, 36% CH_4_, 36% CO_2_ and 20% H_2_O. The performance of the catalyst following poisoning with 4 p.p.m. of H_2_S is shown to illustrate reforming does not occur in absence of an adequate catalyst.

It is important to highlight that this improved coking resistance does not come at the cost of reduced activity for desirable reactions, since these materials show stable catalytic activity throughout long-term reforming test (see Fig. 6) during which deposited Ni particles coke significantly. Indeed, the Ni-based catalyst prepared by exsolution exhibits a level of activity and sensitivity to H_2_S comparable to deposited Ni analogues prepared by conventional deposition methods, but with considerably improved coking resistance^30^.

## Discussion

In this work, we reveal the unique interface developing between the exsolved particles and parent support that leads to at least two emergent effects of great utility: particle pinning and anti-coking behaviour. Thus, redox exsolution may provide an attractive route for tailoring particle–substrate interactions and their subsequent functionality, beyond the capabilities of conventional deposition methods. In particular, the exsolution process may enable the production of socketed metal particles through a single-step reduction treatment. In addition, redox exsolution could be used to deliver such functionality in a selective manner, at a desired location in intricate devices, otherwise inaccessible to deposition methods, by adjusting initial composition at a localized region such as an electrode/electrolyte interface.

We also illustrate some of the key factors that govern exsolution, which may be used for future tailoring of this process, including surface structuring and transport of B-site cations to the surface while highlighting the possible links between them. In particular, we reveal the unexpected role of A-site cations and A-site vacancies in the bulk transport of B-site cations in the perovskite lattice, and hence towards B-site exsolution. Last, the surface structuring occurring in certain perovskites and described here in detail is critical for understanding and interpreting not only exsolution itself, but also the general surface activity and further functionalization of related oxides. A surface consisting of multiple facets and orientations (see, for example, Figs 1b,f and 2) would not only exhibit different catalytic activity from facet to facet depending on the local configuration of atoms, but also particles deposited on such an inhomogeneous surface could exhibit different morphology, distribution and anchorage depending on the polarity and composition of the underlying oxide termination.

## Methods

### Sample preparation and processing

The perovskite oxides were prepared by a modified solid-state synthesis^31^. High purity precursors including La_2_O_3_ (Pi-Kem, >99.99%), TiO_2_ (Alfa Aesar, >99.6%), SrCO_3_ (Aldrich, >99.9%) and Ni(NO_3_)_2_*6H_2_O (Acros, >99%) were used in the appropriate stoichiometric ratios. Certain oxides and carbonates were dried at different temperatures (TiO_2_ and SrCO_3_—300 °C and La_2_O_3_—800 °C) and weighed while warm. The mixture, including nickel nitrate, was quantitatively transferred to a beaker and mixed with acetone and ∼0.05 wt% Hypermer KD1 dispersant. An ultrasonic Hielscher UP200S probe was used into break down agglomerates and homogenize the mixture into a fine, stable dispersion. The acetone was then evaporated at room temperature under continuous stirring and the content of the beaker was quantitatively transferred to a crucible and calcined at 1,000 °C for 12 h to decompose the carbonates and start forming the perovskite phase. The calcined powder was then pressed into pellets and fired at 1,400 °C for 12 h to form the perovskite phase. Porous ceramics, such as the one illustrated in Fig. 1a, were prepared by firing mixtures of as-prepared oxide in powder form with glassy carbon serving as pore-former^31^. Polishing (for example, Fig. 1c) was carried out on a dense (∼94%, polycrystalline) pellet, with a Metaserv 2000 polisher, using initially MetPrep P1200 polishing paper, followed by cloth polishing with MetPrep 6, 3 and 1 μm diamond paste, respectively. Cleaving was carried out on porous pellets, by fracturing the samples with a pestle in a mortar.

To exsolve particles on the surface reduction was carried out in a controlled atmosphere furnace, under continuous flow of 5% H_2_/Ar or pure H_2_, at the temperatures indicated in the main text, with heating and cooling rates of 7 °C min^−1^.

Vapour-deposited Ni (Fig. 5e) was formed using a thermal evaporator (Kurt J. Lesker PRO Line PVD 75), under base pressure of 1.2 × 10^−6^ torr and deposition rate of 0.5 Å s^−1^, followed by annealing in H_2_, at 800 °C, for 4 h.

Sample in Fig. 5a was prepared by infiltration through the following procedure. La_0.4_Sr_0.4_TiO_3_ powder was infiltrated with nickel nitrate aqueous solution at room temperature. After heating in air at 450 °C for 15 min to decompose nickel nitrate, the powder mixture was reduced in dry H_2_ at 500 °C for 5 h. Estimated nickel loading was 2 wt.%.

### Sample characterization

The crystal structure of the prepared perovskites was analysed using powder X-ray diffraction. X-ray diffraction patters were collected at room temperature on a PANalytical Empyrean Diffractometer operated in reflection mode. Selected data were analysed and refined using FullProf software to confirm their crystal structure and determine unit cell parameters (Supplementary Fig. 2). The diffraction peaks were fitted using a pseudo-Voigt profile. Refined parameters include: scale factor, background polynomial parameters or linear interpolation between a set of background points of refinable heights, unit cell parameters, peak profile parameters *u*, *v*, *w* and *η* (Lorentzian/Gaussian distribution), zero shift, atomic positions and site occupancies. A general thermal factor was initially set for the whole pattern and in latter stages refined and eventually converted to atomic isotropic displacement factors for individual atomic sites. The structural information was then used to construct the crystal structure by using Crystal Maker for Windows software (Supplementary Fig. 2b,c).

Nano-E microscope (Pacific Nanotechnology) was used to collect AFM images in tapping mode with silicon tips (Aspire CT300R) from NanoScience Instrument. All AFM image analyses were carried out using Gwyddion software package.

A JEOL JSM-6700 field emission SEM equipped with secondary and backscattered electron detector was used for investigating the surface morphology and phase homogeneity, respectively. All SEM samples were sufficiently conductive; no gold or carbon deposition was required to prevent specimen charging. High-magnification secondary and backscattered electron images were obtained using a FEI Scios electron microscope. Selected micrographs (for example, Fig. 5) were converted to false colour micrographs by colouring the secondary electron image in green, the backscattered analogue in red and then blending them with the help of Mathematica 10 for Windows.

Preparation of a specimen for TEM was carried out using a JEOL JIB-4501 multibeam focused ion beam-scanning electron microscope system. The nickel particles and surface were preserved by the deposition of a protective carbon layer, before milling and thinning using the gallium focused ion beam. High-resolution scanning transmission electron microscopy was carried out on a JEOL ARM 200F. Further electron diffraction characterization was carried out on a JEOL JEM-2010 TEM.

XPS was carried out on a Kratos Axis Ultra-DLD photoelectron spectrometer equipped with an Al monochromatic X-ray source, and the data were analysed using CasaXPS software. The spectra were calibrated based on the C 1*s* peak from adventitious carbon. Quantification was performed based on the area of peaks of interest (La 3*d*_5/2_, Ti 2*p*_3/2,1/2_, Sr 3*d*_5/2,3/2_) after a linear type background subtraction, and the main results summarized in Fig. 1g and Supplementary Table 1. Initially, additional peaks were used for quantification for each chemical species to ensure that the calculated stoichiometry does not depend significantly on different core levels. Ti^3+^ quantification was carried out by fitting the Ti 2*p*_3/2_ peak with individual components.

Particles on flat oxide surfaces were analysed using ImageJ software according to the following procedure. SEM images of adequate magnifications were selected and their contrast and sharpness slightly increased. These were then imported into ImageJ software where particles were outlined based on contrast and distances (in pixels) were calibrated based on the corresponding SEM image scale (in nm). Built-in ImageJ functions were then used to calculate the area of particles based on the number of pixels contained within their set boundaries. This area was assumed to correspond to the average area of the equatorial circle of the spheroid-like particles and thus was used to estimate their average size and further particle size distribution.

### Coking and reforming experiments

The coking test was carried out at 800 °C by flowing 20% CH_4_/H_2_ without humidification. The two gases were mixed right before the reactor inlet.

For the reforming test, the catalyst bed was 3mm deep, and had an internal diameter of 20 mm. Gas hourly space velocity was 19,000 h^−1^. The gas contained 16.5 ml min^−1^ Ar as an internal standard, while the total input gas flow was 300 ml min^−1^.

## 

## Additional information

**Accession codes:** The research data supporting this publication can be accessed at http://dx.doi.org/10.17630/2CA88024-4A83-4690-B66F-B63B98A052D1,

**How to cite this article:** Neagu, D. *et al*. Nano-socketed nickel particles with enhanced coking resistance grown *in situ* by redox exsolution. *Nat. Commun.* 6:8120 doi: 10.1038/ncomms9120 (2015).

## Supplementary Material

Supplementary Information Supplementary Figures 1-15, Supplementary Table 1, Supplementary Note 1 and Supplementary References
