# Supplementary material for: Nano-socketed nickel particles with enhanced coking resistance grown in situ by redox exsolution
Source: Nat Commun. 2015 Sep 11;6:8120. doi: 10.1038/ncomms9120 (PMC4579408; doi:10.1038/ncomms9120)
Supplement: Supplementary Information — Supplementary Figures 1-15, Supplementary Table 1, Supplementary Note 1 and Supplementary References [file ncomms9120-s1.pdf]

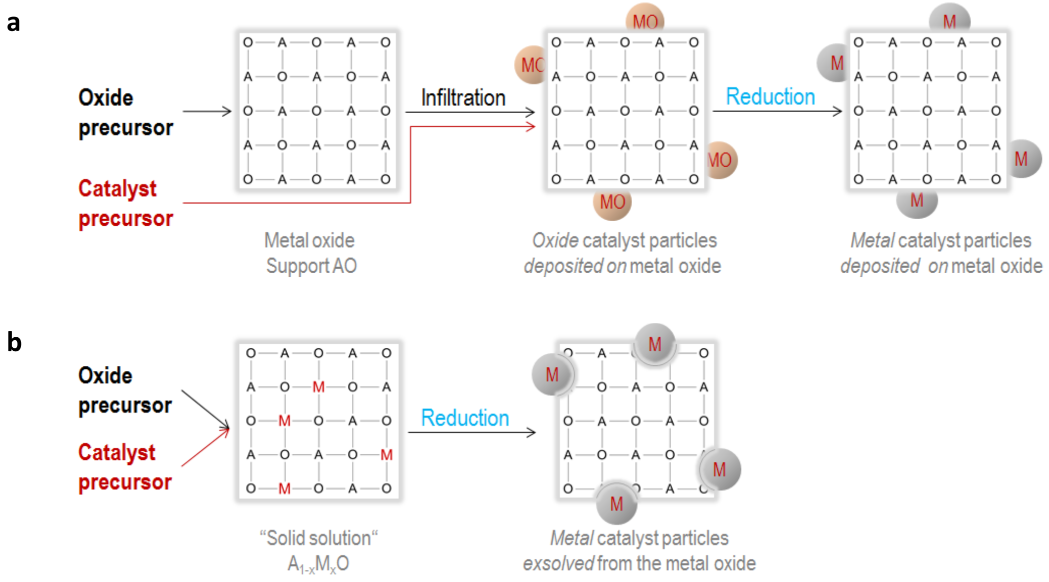

Supplementary Figure 1 | Comparative illustration of the steps required to decorate an oxide support AO with catalyst particles M through chemical infiltration or in situ redox exsolution. (a) chemical infiltration usually requires several deposition and thermal treatment steps followed by a reduction to produce the metal particles M on the outer surface of the support AO. (b) in *in situ* redox exsolution M is incorporated in its cation form in the crystal lattice of AO and exsolved in one step, upon reduction.

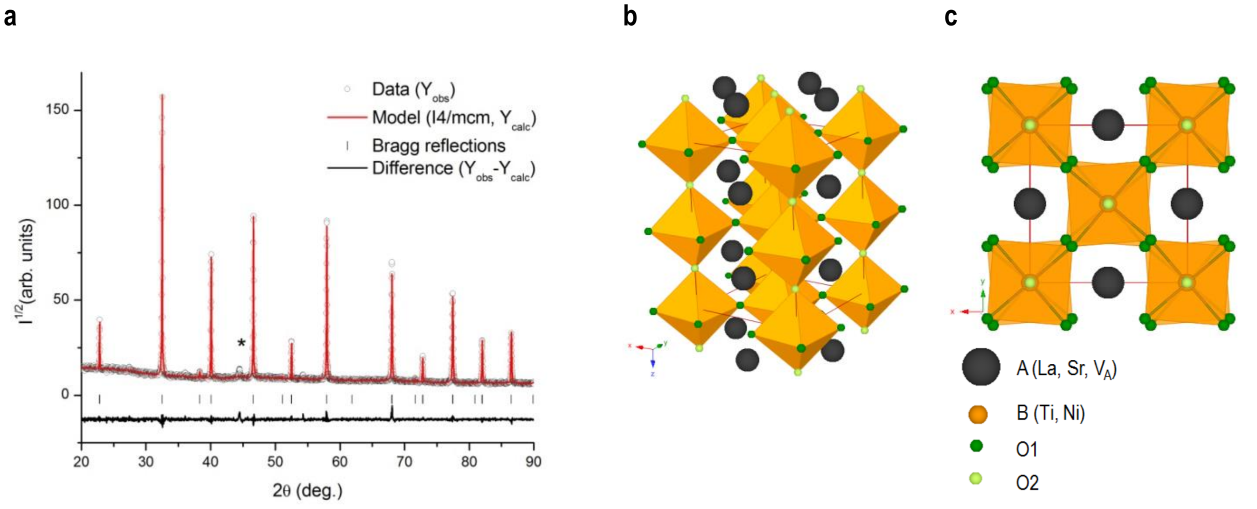

Supplementary Figure 2 | Rietveld refinement and corresponding crystal structure of $La_{0.4}Sr_{0.4}Ni_{0.03}Ti_{0.97}O_{3-\gamma}$. (a) Rietveld refinement of the room temperature powder XRD pattern using I4/mcm ($a^0a^0c^-$) space group, a = b = 5.5067 Å, c = 7.7852 Å. R_p = 6.67%, R_{wp} = 8.82%, R_e = 7.77%, χ^2 = 1.29 (b) Three-dimensional overview of the crystal structure. (c) Projection along the z axis showing out of phase tilting along this axis.

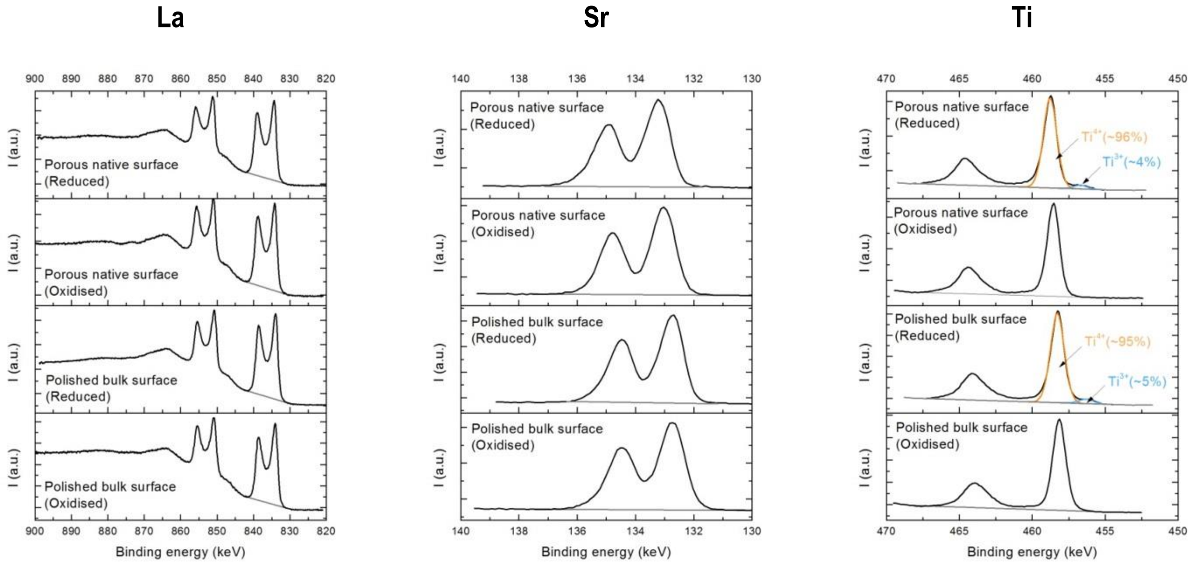

Supplementary Figure 3 | XPS spectra of La, Sr and Ti for the samples indicated in Fig. 1a, b, c, d. Black lines represent experimental data, grey lines the background, orange lines Ti^{4+} peak fit, and blue lines Ti^{3+} peak fit.

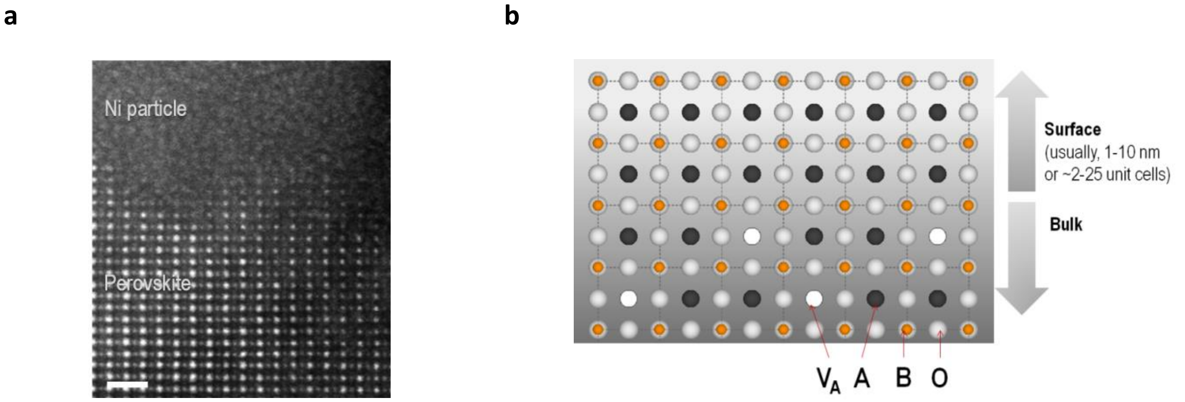

Supplementary Figure 4 | (a) TEM image of the native surface of $La_{0.4}Sr_{0.4}Ni_{0.03}Ti_{0.97}O_{3-\gamma}$ similar to the one displayed in Fig. 1b, in the vicinity of an exsolved particle; scale bar 1 nm. (b) Schematic atomic model of the structure of $La_{0.4}Sr_{0.4}Ni_{0.03}Ti_{0.97}O_{3-\gamma}$ going from bulk to the native surface based on the XPS surface stoichiometry presented in Fig. 1a, b.

a b c

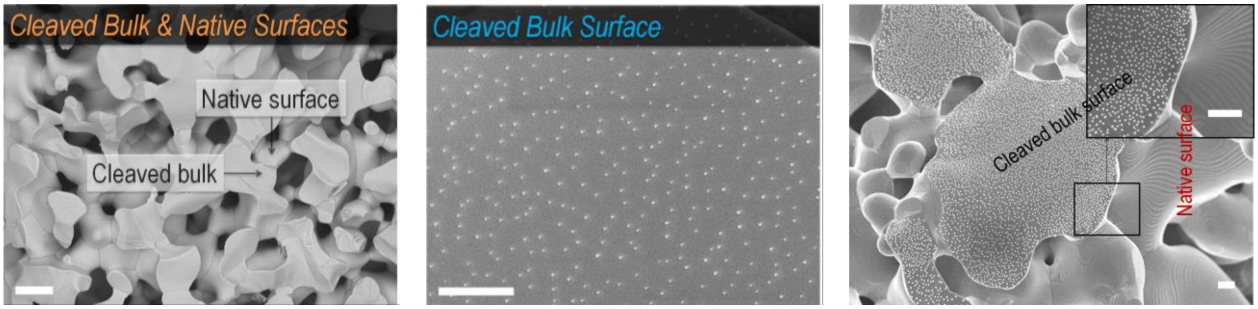

Supplementary Figure 5 | (a) Cleaved porous $La_{0.52}Sr_{0.28}Ni_{0.06}Ti_{0.94}O_3$ sample revealing bulk-like surfaces alongside the already-present native surfaces; scale bar, 20 µm. (b) detail from the cleaved bulk surface region of a $La_{0.4}Sr_{0.4}Ni_{0.03}Ti_{0.97}O_{3-\gamma}$ sample with microstructure similar to sample (a) after reduction (5% H_2/Ar, 900 °C, 20 h); scale bar, 1 µm. (c) detail of sample (a) after reduction (5% H_2/Ar, 900 °C, 12 h) showing no particles grow on the native surfaces in this case, although numerous particles grow on the cleave surface; scale bars, 1 µm.

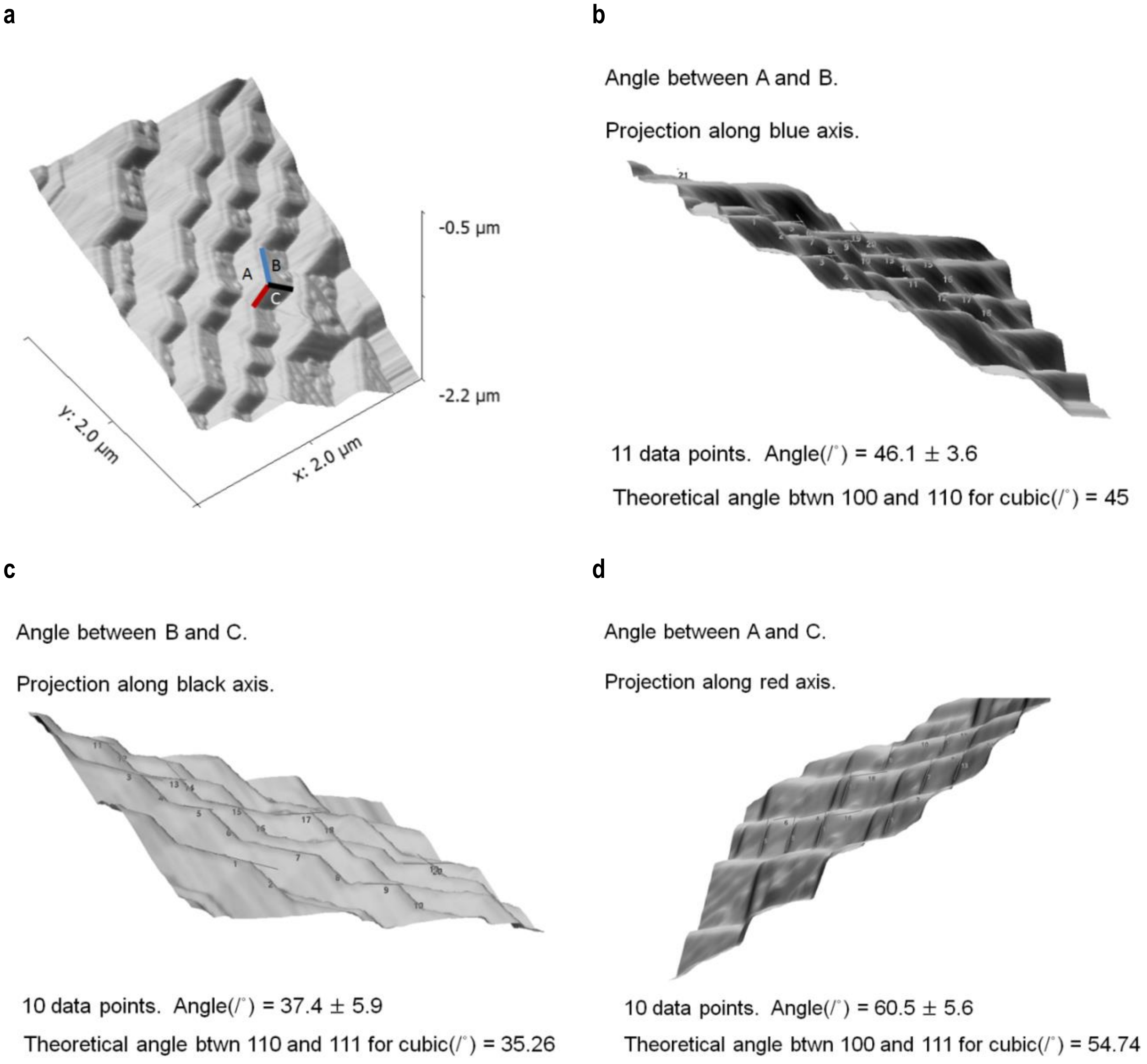

Supplementary Figure 6 | Facet angle measurements by 3D AFM surface reconstruction for the native surface shown in Fig. 1b. The reconstructed 3D image is shown in Fig. 1e.

a **b** **c**

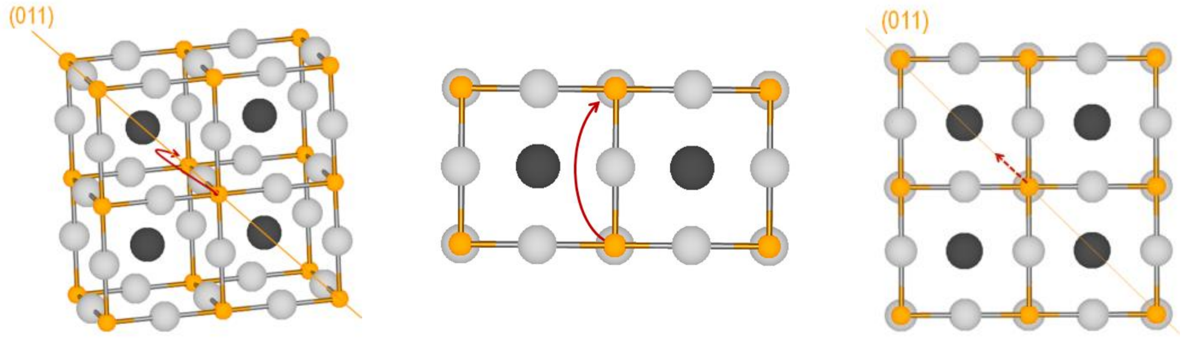

Supplementary Figure 7 | B-site cation diffusion in perovskites. (a) 3D view of the curved migration path in the (011) plane. (b) Projection from the top, [001]. (c) Projection in the direction of diffusion, [100]. Adapted from reference [1].

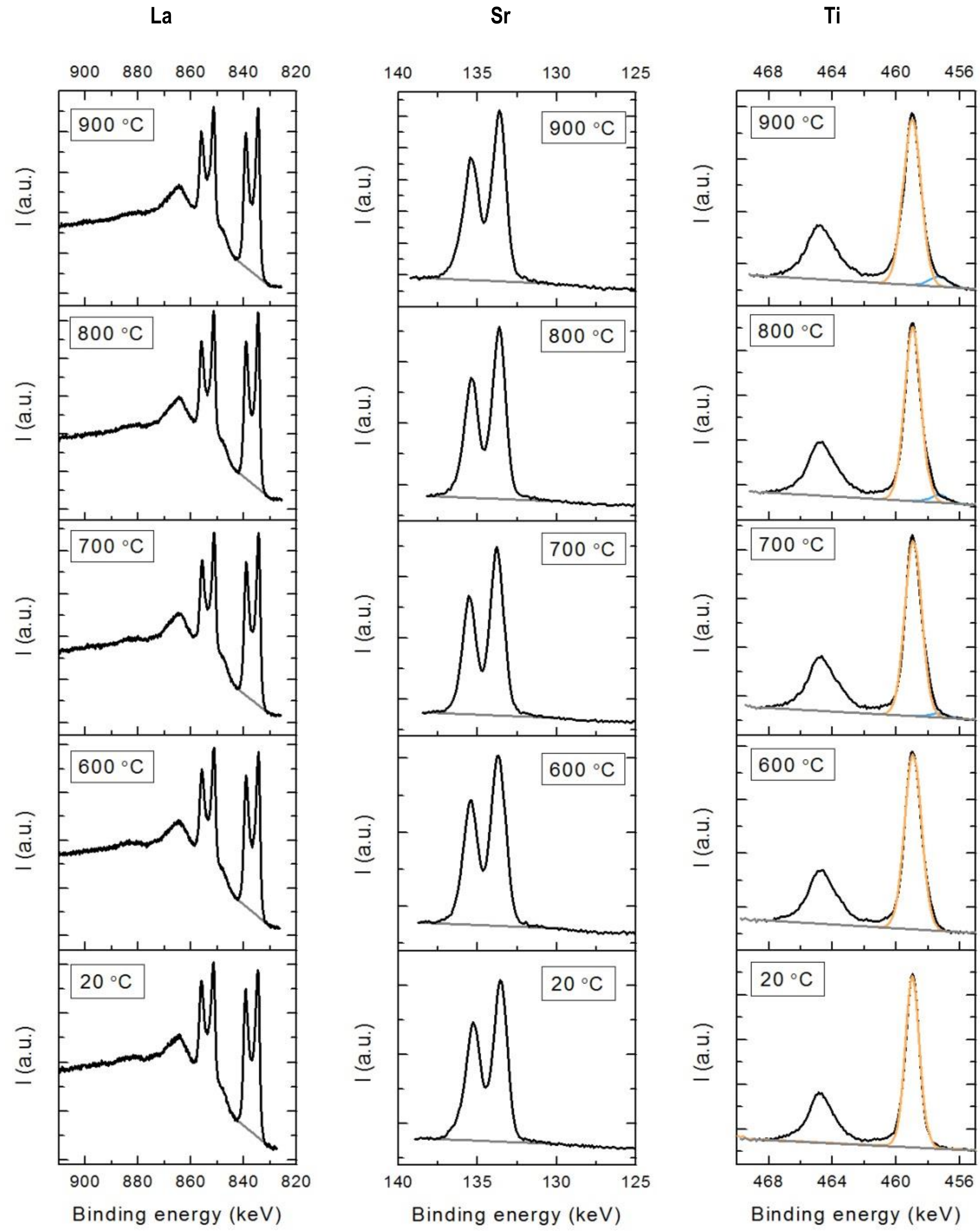

Supplementary Figure 8 | XPS spectra corresponding to Fig. 1g. Black lines represent experimental data, grey lines the background, orange lines Ti^{4+} peak fit, and blue lines Ti^{3+} peak fit.

a

b

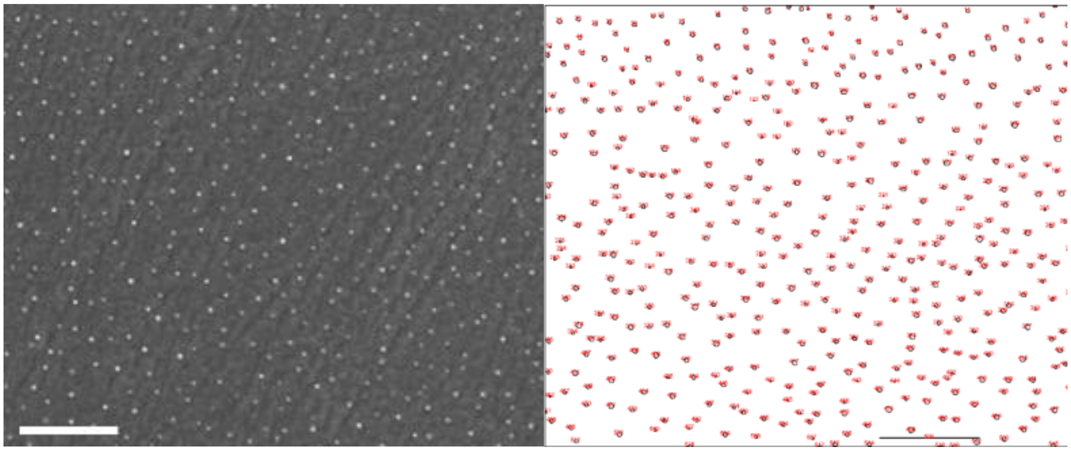

c

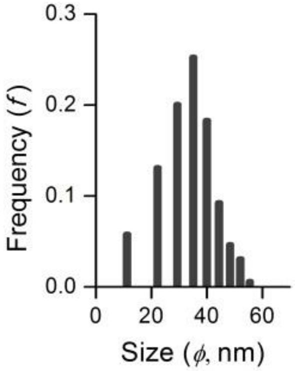

Supplementary Figure 9 | Particle analysis on the surface shown in Fig. 1d. (a) SEM image corresponding to Fig. 1d. (b) Ni particle mapping and contour extracted with ImageJ from Supplementary Fig. 9a. (c) Particle size distribution calculated with ImageJ based on Supplementary Fig. 9b. Scale bars, 1 μm.

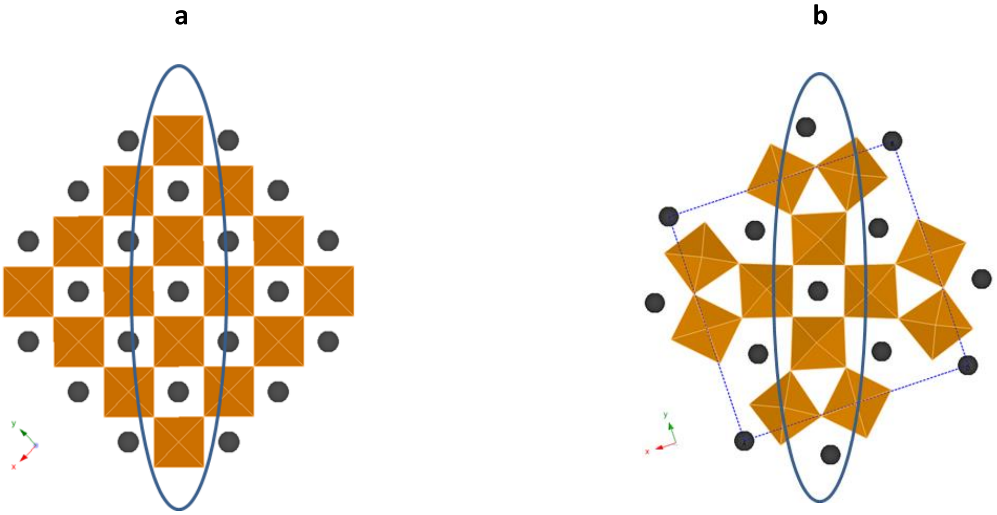

Supplementary Figure 10 | Comparison between (a) normal perovskite crystal structure and (b) projection down c of a hexagonal tungsten bronze (a high A-site deficient variant of the perovskite lattice) showing units coupling A-site vacancies with the smaller La^{3+} ion through a B site octahedron. This suggests a possible defect unit that could potentially couple La, Ni and an A-site vacancy consistent with the cooperative movement of La and Ni as tungsten bronzes.

a b

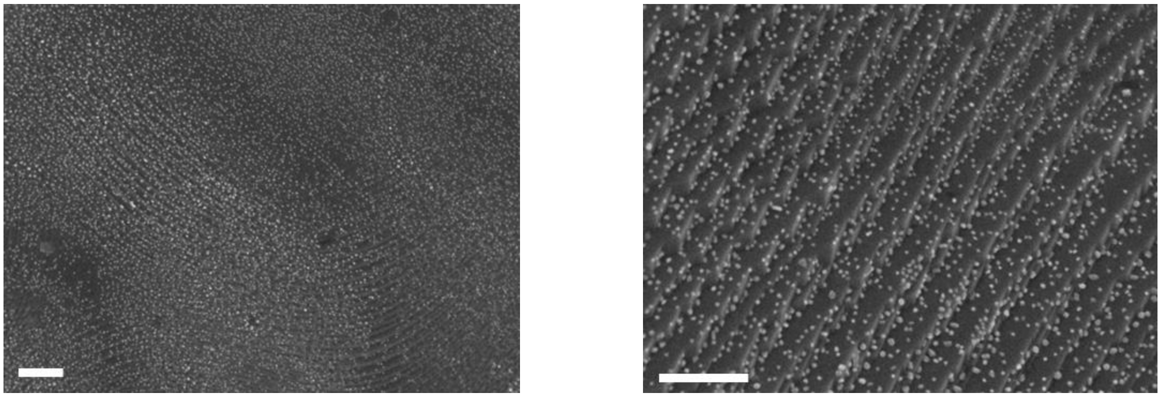

Supplementary Figure 11 | Exsolved Ni particles from the native surface of $La_{0.46}Sr_{0.34}Ni_{0.03}Ti_{0.97}O_3$ after reduction in pure H_2 at 900 °C for 2.5 hours. Scale bars, 1 μm.

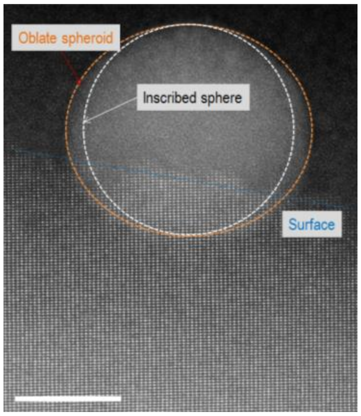

Supplementary Figure 12 | Particle shape and embedding highlighted on the TEM image shown in Fig. 3a. Scale bar, 10 nm.

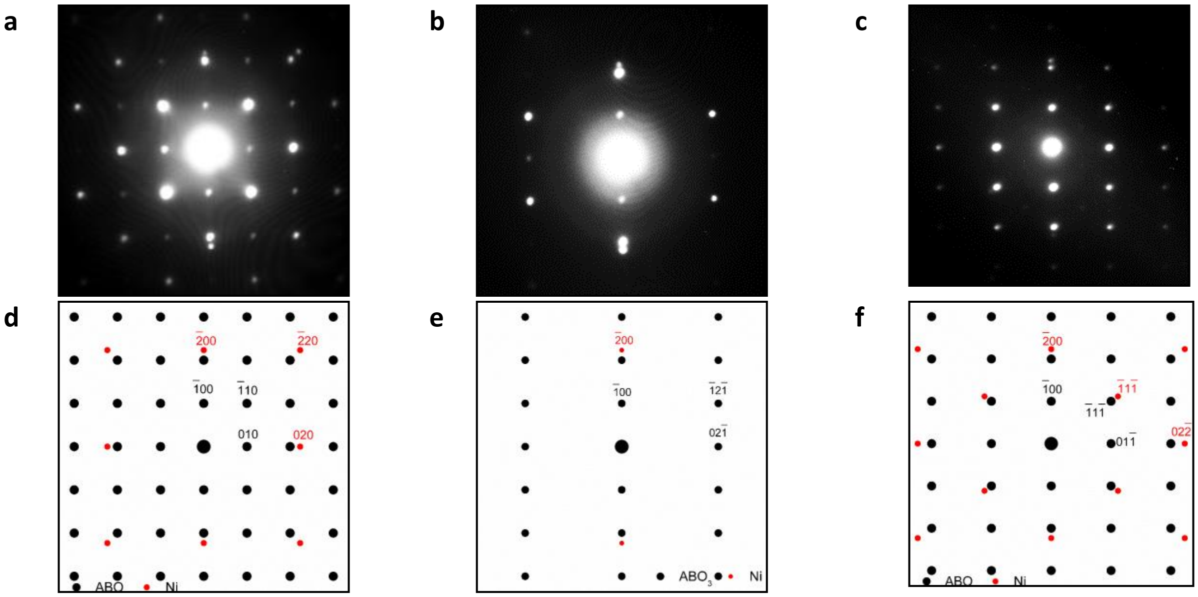

Supplementary Figure 13 | Orientation relationship between exsolved particles and parent perovskite for the sample in Fig. 3a. (a), (b) and (c) show selected area electron diffraction (SAED) patterns containing reflections from the perovskite and a nickel particle. The patterns correspond to the [001], [112] and [011] zone axes of the perovskite oxide, in each case nickel reflections corresponding to the same zone axis are also visible, as shown in (d), (e), (f), respectively. The presence of these reflections is consistent with the orientation relationship $[010]_P//[010]_{Ni}$ $(001)_P//(001)_{Ni}$ which we have previously observed in other system we investigated, but which have higher Ni substitution level ($La_{0.8}Ce_{0.1}Ti_{0.6}Ni_{0.4}O_3$, see reference [2]), perhaps suggesting that exsolution generally occurs epitaxially with respect to the parent perovskite. However, in this case, not all expected nickel reflections are visible. The absence of these reflections can be attributed to the very small volume of the nickel particles, the absence of higher intensity low index reflections close to the centre of the diffraction pattern due to the face centred cubic structure of nickel and the possibility of variation from the ideal orientation relationship. Of the eight to ten nickel particles present in the electron transparent region of the TEM sample, reflections consistent with this orientation relationship could only be obtained for two. Whether this is due to beam damage from imaging or sample preparation or the absence of this specific orientation relationship could not be determined.

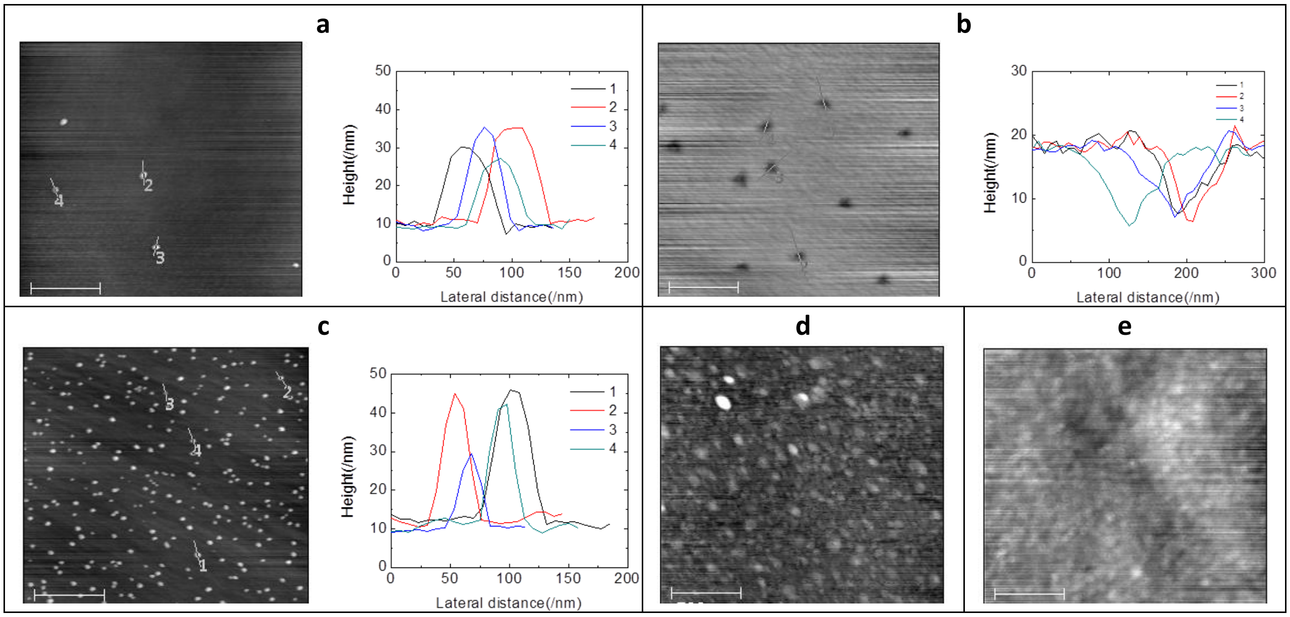

Supplementary Figure 14 | AFM images and height profiles of: (a) Ni particles exsolved from $La_{0.4}Sr_{0.4}Ti_{0.97}Ni_{0.03}O_{3-\delta}$ (900 °C, 5 h, dry H_2); (b) sample (a) after etching (HNO_3) at room temperature for 20 minutes; (c) Ni particles from Ni nitrate solution deposition (900 °C, 6 h, dry H_2); (d) Sample (c) after etching at room temperature overnight showing now obvious pits; (e) further etching at 70 °C for 2 hours. Scale bars, 500 nm.

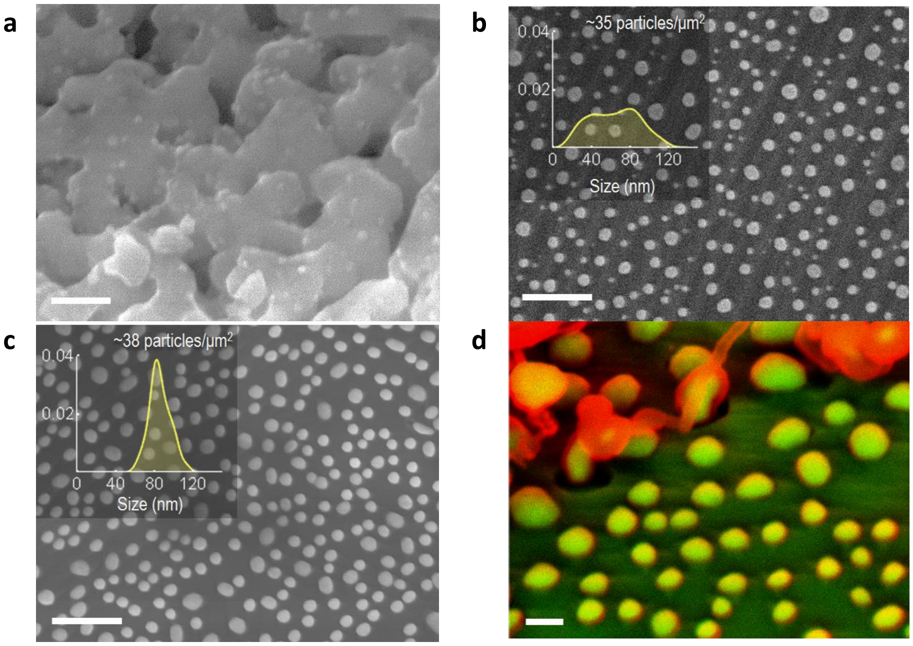

Supplementary Figure 15 | (a) ~20 nm Ni particles formed by infiltration on $La_{0.4}Sr_{0.4}TiO_3$. (b) 30-100 nm Ni particles prepared by vapour-deposition on $La_{0.4}Sr_{0.4}TiO_3$ (the inset shows the particle size distribution). (c) 80-90 nm size Ni particles exsolved from $La_{0.52}Sr_{0.28}Ni_{0.06}Ti_{0.94}O_3$ (5% H_2/Ar, 1000 °C, 12 h; the inset shows the particle size distribution). (d) False colour micrograph of sample (c) after coking experiment showing particle uplifting which leaves behind corresponding empty sockets. Scale bars, 1 μm (b, c), 100 nm (a, d).

Supplementary Table 1 | Quantification of the XPS spectra in Supplementary Fig. 8 which was used to derive the data plotted in Fig. 1g. Peak areas are given in arbitrary units as calculated by CasaXPS software and also include transmission function and mean free path contributions. RSF represents the corresponding relative sensitivity factor.

	**Temperature (°C)**	**20**	**600**	**700**	**800**	**900**
**La $3d_{5/2}$**	Peak area (a.u.)	6969.6	6832.1	7758.3	8888.2	8172.9
	RSF	5.473	5.473	5.473	5.473	5.473
	Peak area/RSF (a.u.)	1273.5	1248.3	1417.6	1624.0	1493.3
	**Atomic %**	**35.2**	**34.9**	**35.4**	**36.8**	**38.2**
**Sr $3d_{5/2,3/2}$**	Peak area (a.u.)	1261.5	1258.3	1372.7	1426.5	1172.5
	RSF	1.843	1.843	1.843	1.843	1.843
	Peak area/RSF (a.u.)	684.5	682.7	744.8	774.0	636.2
	**Atomic %**	**18.9**	**19.1**	**18.6**	**17.6**	**16.3**
**Ti $2p_{3/2,1/2}$**	Peak area (a.u.)	3321.7	3283.2	3676.5	4026.3	3561.4
	RSF	2.001	2.001	2.001	2.001	2.001
	Peak area/RSF (a.u.)	1660.0	1640.8	1837.3	2012.1	1779.8
	**Atomic %**	**45.9**	**45.9**	**45.9**	**45.6**	**45.5**
**Atomic ratios**	**La/Ti**	**0.77**	**0.76**	**0.77**	**0.81**	**0.84**
	**Sr/Ti**	**0.41**	**0.42**	**0.41**	**0.38**	**0.36**
	**(La+Sr)/Ti**	**1.18**	**1.18**	**1.18**	**1.19**	**1.20**
**Ti^{4+} $2p_{3/2}$**	Peak area (a.u.)	2032.2	2022.0	2214.0	2396.2	2099.0
	RSF	2.001	2.001	2.001	2.001	2.001
	Peak area/RSF (a.u.)	1015.6	1010.5	1106.4	1197.5	1049.0
**Ti^{3+} $2p_{3/2}$**	Peak area (a.u.)	0.0	19.0	57.4	110.2	135.0
	RSF	2.001	2.001	2.001	2.001	2.001
	Peak area/RSF (a.u.)	0.0	9.5	28.7	55.1	67.5
	**$Ti^{3+}/(Ti^{3+}+Ti^{4+})$**	**0.00**	**0.01**	**0.03**	**0.04**	**0.06**

Supplementary Note 1

The average number of Ni atoms contained by the particles shown in Supplementary Fig. 9a, calculated based on the particle distribution shown in Supplementary Fig. 9d is given by:

$$N_{Ni,p} = \frac{\rho_{Ni}}{A_{Ni}} \cdot N_A \cdot \frac{\pi}{6} \cdot \sum_i (f_i \cdot \phi_i^3) \approx 1.19 \cdot 10^9 Ni\ atoms \quad (1)$$

Where: ρ_{Ni} is Ni metal density, A_{Ni} is the atomic weight of Ni, N_A is Avogadro's number, f_i is the fraction of particles having diameter ϕ_i according to Supplementary Fig. 9d.

The average number of Ni atoms in a parallelepiped perovskite volume of length L, width W and depth d is given by:

$$N_{Ni} = \frac{L \cdot W \cdot d}{a_p^3} \cdot x_{Ni} \quad (2)$$

Where a_p is the pseudo-cubic perovskite unit cell parameter and x_{Ni} is Ni stoichiometry in the perovskite.

The average depth of a virtual perovskite parallelepiped containing $N_{Ni,p}$ atoms over an area equal to the one in the image shown in Supplementary Fig. 9a:

$$d = \frac{N_{Ni,p} \cdot a_p^3}{L \cdot W \cdot x_{Ni}} \approx \frac{1.19 \cdot 10^9 \cdot (0.39\ nm)^3}{5950 \cdot 4475\ nm^2 \cdot 0.03} \approx 88\ nm \quad (3)$$

In conclusion, for the micrograph shown in Supplementary Fig. 9a, Ni atoms appear to be depleted from ~88 nm deep beneath the surface in order to account for the amount of Ni observed on the surface in the form of metal particles.

The average number of La atoms in a parallelepiped perovskite volume of length L, width W and depth h is:

$$N_{La} = \frac{L \cdot W \cdot h}{a_p^3} \cdot x_{La} \quad (4)$$

Where x_{La} is La stoichiometry in the perovskite. The number of La atoms enriching the surface during reduction through diffusion from the bulk, over an area equal to the one in Supplementary Fig. 9a is given by:

$$N_{La} = \frac{L \cdot W \cdot h}{a_{p,RED}^3} \cdot x_{La,RED} - \frac{L \cdot W \cdot h}{a_{p,OX}^3} \cdot x_{La,OX} \approx \frac{L \cdot W \cdot h}{a_p^3} \cdot (x_{La,RED} - x_{La,OX}) \quad (5)$$

Where $x_{La,RED}$ is La surface stoichiometry in the perovskite after reduction (from XPS, Fig. 1d) and $x_{La,OX}$ is La surface stoichiometry in the perovskite prior to reduction (from XPS, Fig. 1a), also assuming the surface is $h = 10\ nm$ thick (typical XPS detection depth):

$$N_{La} = \frac{5950 \cdot 4475 \cdot 10\ nm^3}{(0.39\ nm)^3} \cdot (0.61 - 0.38) = 1.03 \cdot 10^9\ La\ atoms \quad (6)$$

In conclusion, the amount of Ni metal contained in the surface particles shown in Supplementary Fig. 9a, $\sim 1.19 \cdot 10^9$ Ni atoms, is of the same order of magnitude as the number of La atoms that diffused from the bulk, $\sim 1.03 \cdot 10^9$ La atoms.

Supplementary references

1. De Souza, R. A., Islam, M. S. & Ivers-Tiffée, E. Formation and migration of cation defects in the perovskite oxide LaMnO3. *J. Mater. Chem.* **9,** 1621–1627 (1999).
2. Neagu, D., Tsekouras, G., Miller, D. N., Ménard, H. & Irvine, J. T. S. In situ growth of nanoparticles through control of non-stoichiometry. *Nat. Chem.* **5,** 916–923 (2013).
